# The Effectiveness of Lee Silverman Voice Treatment (LSVT LOUD) on Children’s Speech and Voice: A Scoping Review

**DOI:** 10.3390/brainsci14090937

**Published:** 2024-09-19

**Authors:** Angelos Papadopoulos, Louiza Voniati, Nafsika Ziavra, Dionysios Tafiadis

**Affiliations:** 1 School of Health Rehabilitation Sciences, University of Patras, 26500 Patras, Greece; angelospapadopoulos@gmail.com; 2 General Children’s Hospital of Patras “Karamandaneio”, 26331 Patras, Greece; 3 Department of Health Sciences, Speech and Language Therapy, European University, Nicosia 22006, Cyprus; l.voniati@euc.ac.cy; 4 Department of Speech and Language Therapy, School of Health Sciences, University of Ioannina, GR45500 Ioannina, Greece; nziavra@uoi.gr

**Keywords:** Lee Silverman Voice Treatment, LSVT LOUD, children, speech, voice, scoping review

## Abstract

Background: This scoping review had as a primary goal a review of the literature and the an analysis of the possible effectiveness of the LSVT LOUD approach in children with voice and speech deficits. Methods: A search was conducted in the Scopus and PubMed databases in May of 2024. Eleven articles were obtained from the search. The standards of PRISMA recommendations were used for scoping reviews and the PCC framework was used for the eligibility criteria. Furthermore, the study used the instructions in the Cochrane Handbook for a quality assessment. The Mendeley Reference Manager software collected the studies and removed duplicates. Results: The reviewed studies employed formal and informal measures to assess voice and speech abilities in the children. Regarding the sample’s characteristics, the studies mostly included children with Cerebral Palsy (CP) and also those with Down Syndrome (DS). All the studies reported that children with CP and DS undertook a total dose of the LSVT LOUD treatment. Significant post-treatment findings indicated increased speech function and sound pressure level, regarding the auditory–perceptual ratings of voice and speech improvement. In many studies, parents’ and expert listeners’ ratings of voice, perception of vocal loudness, speech, and communication indicated improvement. Conclusions: The majority of the included studies provide positive evidence for the LSVT as an approach. However, the small sample size that featured in the studies, as well as their limitations, made these conclusions uncertain. Moreover, the study’s findings provided recommendations that speech language therapists and other clinicians need to follow when setting a treatment plan with children with CP and DS.

## 1. Introduction

Numerous studies have documented the frequency of voice disorder in preschool- [1,2] and school-aged children [3,4,5,6,7]. Additionally, a variety of studies report prevalence rates in terms of different countries [3,5,6] and cultural backgrounds [1]. In general, the prevalence of children’s Voice Disorders (chVD) ranges from 1.4% to 6.7% [1,7]. Furthermore, many studies have documented the prevalence of chVD and other speech and language disorders [5,6] and in relation to specific medical conditions such as cerebral palsy (CP) and down syndrome (DS). Approximately 40% to 90% of children diagnosed with CP experience motor speech impairments, which have a detrimental impact on their ability to communicate clearly [8,9]. In addition, the motor speech characteristics of children with DS have traditionally been classified as childhood dysarthria [10]. Specifically, 37.8% had childhood dysarthria, and the dysarthria subtype that was most commonly observed was ataxia. As indicated above, dysarthria is common in children with CP and DS diagnosis. Accordingly, the necessity of screening and assessment procedures [4,11], as well-documented therapy for chVD, is of high priority for speech-language pathologists [12,13].

Regarding children with CP, the literature [14] has reported that their vocal quality is altered. These changes refer to a reduced ability to control loudness and an altered voice quality [14,15,16], and, according to Nordberg et al., those who listen to the voices of children with CP rated them as having a strained–strangled, harsh, wet hoarseness [15]. Something that was noticed by a study [17] is that the voice quality of these children differs, and many times changes are present during a part of a sentence rather than there being consistency throughout the sentence. Furthermore, the voice quality differences result from motor control deficits of the laryngeal subsystem and/or coordination of the laryngeal and respiratory subsystems [14]. Specifically, a study concluded that children have presented vocal quality issues, and their vocal quality is characterized as constricted and creaky as a result of lower spectral tilt and greater noise. Worse vocal fold articulation was also demonstrated when producing single vowels, and low vowels produced less constriction compared to high vowels.

The children with DS also exhibited vocal quality issues, such as harsh, husky, monotonous, low-pitched, and ventricular voices [18,19]. These are a result of the squeezing of the larynx during phonation [18]. Moreover, children with DS have increased rates of laryngeal issues when compared to the population of children without DS [20]. The literature [20,21] has reported that these children are at a higher risk of subglottic stenosis, vocal fold paralysis, and laryngomalacia, and that the most common etiology of dysphonia in children with DS is vocal fold immobility and hypomobility. 

In recent research, there has been a growing interest in treating pediatric voice disorders [22]. Voice therapists and researchers are aware that many of the well-established adult voice treatment techniques can be adjusted to accommodate children’s developmental stages, but there is still much to learn about the essential elements and techniques needed for positive results [22,23], as well as several factors (e.g., parental perceptions of chVD, patient demographic data, etc.), such as the duration of therapy [12,24], that have been set as barriers to a successful outcome of voice therapy (VT) [25]. In their study, Braden and Thibeault (2020) concluded that even though voice therapy is a gold standard and established as the intervention for harmless tumors in adults, it has become more widely recommended for children as well. They have also underlined the fact that there is still debate, as in other studies [12,25,26], regarding the effectiveness of therapy for children with harmless tumors. There is conflict about what therapy should consist: only of vocal hygiene instruction [27], or whether it should also incorporate a direct intervention [23]. As an outcome of their study, they stated that children with benign vocal fold lesions and dysphonia following voice therapy had statistically significant improvements in phonation cut-off pressure and the perceptual quality of the voice. They also suggested that voice therapy is beneficial for enhancing children’s voice quality [23], which was in agreement with other studies as well [12]. 

VT is divided into direct and indirect interventions and can be delivered via extrinsic and/or intrinsic approaches [28,29,30,31]. These interventions are organized in detailed programs/protocols with specific therapy tasks or components [28]. The seven well-known VT protocols are as follows: (i) Manual Circumlaryngeal Therapy (MCT) [32,33], (ii) Laryngeal Manual Therapy (LMT) [34,35], (iii) Vocal Function Exercises (VFE) [36,37], (iv) Resonant Voice Therapy (RVT) [38,39], (v) Accent Method [40,41,42], (vi) Confidential Voice Therapy (CVT) [43], and (vii) the Lee Silverman Voice Treatment (LSVT) [44,45]. 

Dr. Lorraine Ramig developed the LSVT method which has been a research focus for over 25 years [44,45]. LSVT has the scope to increase vocal fold adduction and loudness. This could be achieved by high-effort, intensive stimulation [46]. The literature features several randomized control trials [45,47,48,49] which have proved that LSVT can improve vocal intelligibility, speech issues, and quality of life [50]. Currently, LSVT has standardized research methods, and the reliability of outcome indicators has improved [46,47,51,52]. 

LSVT uses a simple but intensive approach while promoting a healthy increase in vocal loudness. The aforementioned indicated improvements are not only in voice production but also in speech articulation and intelligibility [50]. Studies have reported that LSVT LOUD positively affects swallowing, facial expression, and neural function, as revealed by PET [50,53,54] in people with PD. It has been implemented in individuals with Parkinson’s, multiple sclerosis, ataxia, traumatic brain injury, DS, and CP. The implementation was made without modifying the primary treatment protocol [50]. LSVT trains Parkinson’s disease (PD) patients at moderate stages of the disease and other atypical Parkinsonisms to employ their vocal cords at a more normal loudness in different settings. The treatment is delivered via several sessions under the guidance of a certified SLP four times a week for four weeks [55].

The LSVT method was also applied in children with voice and speech disorders [56,57,58,59]. According to Fox and Boliek’s (2012) study, which examined the impact of LSVT on children with dysarthria and spastic cerebral palsy (CP), their preliminary data showed that children with spastic CP not only were able to deal with accurate voice treatment but also demonstrated improvement in a few specific areas of vocal functioning. Another study by Langlois et al. (2020) evaluated the treatment effects of LSVT on (1) vowel duration, (2) acoustic vowel space, and (3) the ratio of F2/i/ to F2/u/, which were analyzed using acoustic data from children with secondary motor speech disorders. The authors concluded that it may foster articulation improvements for children with CP changes, indicated in the duration of vowels and acoustic vowel space, and for children diagnosed with DS, observed in alterations in acoustic vowel space. Furthermore, Reed et al. (2017), in their research on eight children with motor speech disorders and CP, compared to an age- and sex-matched group of peers whose speech has developed without motor speech disorders and CP, administered LSVT in a 12-week program to examine any changes in white matter integrity. The researchers implied that the long-term practice of skills during therapy improved the integrity of the white matter neural networks of speech production.

Finally, Boliek et al. (2022), in their phase I outcomes for LSVT for children with Down syndrome, suggested that phase II treatment trials employing LSVT with a bigger population of children with DS will set the foundation for the method of obtaining treatment evidence data. They have also reported that their early findings demonstrated some significant therapeutic improvements in children with DS as well those not experiencing any high levels of intolerance.

To our knowledge, this could cover a research gap in the literature regarding the lack of such a review in pediatric samples. LSVT has been implemented and standardized in several randomized clinical trial studies in adult samples over the years [46]. Until now, there have only been systematic reviews of adults that systematically compared the effectiveness of LSVT with speech therapy or with no intervention for dysarthria in the adult population [46,60]. Given what has just been mentioned, the current scoping review aims to explore and analyze the literature on the LSVT LOUD approach’s effect on diagnosis for children with CP and DS with voice and speech problems arising from dysarthria secondary to CP and DS. 

### The Aim of the Study

This scoping review was conducted to enhance the current body of literature by addressing the following research questions: is the LSVT LOUD approach effective and practical for children regarding their voice and speech issues that arise from various disorders?

Moreover, the primary objective of this scoping review is to supply researchers with an up-to-date and complete information resource on the subject, promoting its use in clinical practice. Moreover, it would be considered a valuable manuscript since it consisted of useful pieces of the literature. In addition, this scoping review provides a comprehensive view of the impact of the LSVT LOUD approach in speech and voice treatment.

## 2. Materials and Methods

### 2.1. Databases and Search 

The authors conducted the research in the Scopus and PubMed databases in May 2024. The articles were selected from relevant journals. The following keywords were used in both databases: “LSVT”, “LOUD”, and “CHILDREN”. Following the search, the researchers read the abstracts to verify that the studies included the LSVT LOUD implementation in children. Specifically, after we completed searches of the literature, and identified studies, we conducted screening of the titles and abstracts of each article to determine if they met the criteria for inclusion. These criteria for titles and abstracts were pre-defined and were as follows:

- Date range of publication: the dataset for this study included all available literature without any time restrictions.
- Study design type: only research articles and no review studies.
- Study focuses on a specific disease, condition, or patient population: only children.
- Study focuses mainly on a specific intervention: LSVT LOUD
- Study took place in a certain country/hospital or other context: no restriction of the context.
- Study written in a specific language: we only included studies written in English. However, all the available studies were written in English.

**Figure 1:**
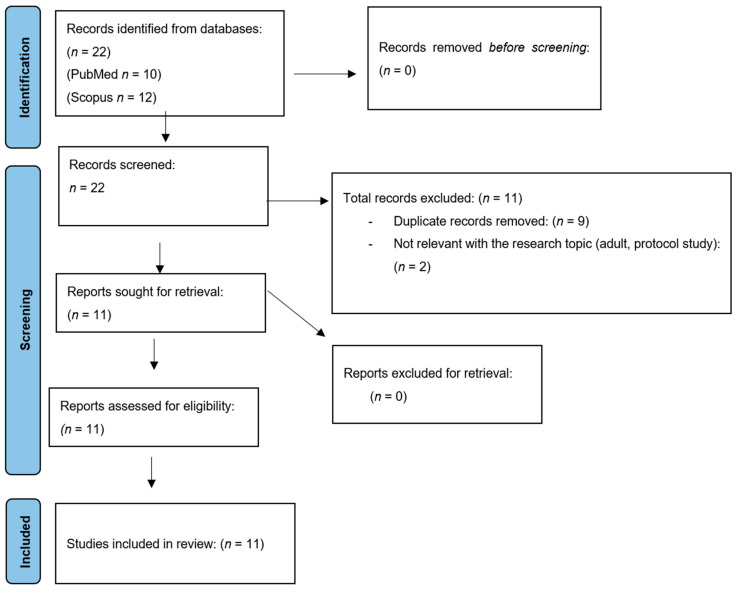
PRISMA ScR flow diagram.

Furthermore, Figure 1 shows the process flow diagram for this scoping review, which is based on the standards of PRISMA guidelines for scoping reviews (PRISMA-ScR) [61]. Moreover, the researchers used the PCC framework as guidance to construct the criteria for this scoping review (Table 1) [62].

The electronic research strategy for Scopus was as follows: (TITLE-ABS-KEY (LSVT LOUD) AND TITLE-ABS-KEY (CHILDREN)), and for PubMed was as follows: ((LSVT) AND (LOUD)) AND (CHILDREN). 

### 2.2. Eligibility Criteria

The current review included only articles that met specific qualifying criteria. The criteria were as follows: the article (1) investigates children treated with the LSVT LOUD approach, (2) focuses on addressing deficiencies in speech and voice issues, (3) is written in the English language, (4) utilizes either standardized or non-standardized measures for the speech and voice assessment process, (5) is only a research article, with no review studies allowed, and that (6) every county/hospital or other place would be included with no restriction of the context.

On the other hand, the criteria for exclusion were as follows: (1) data such as letters to the editor or other review studies were excluded, and (2) studies that were part of a thesis or dissertation (grey literature) were excluded.

The database screening resulted from 12 Scopus and 10 PubMed abstracts that were reviewed to confirm if they met the inclusion criteria for the scoping review study. At this point, it needs to be mentioned that two (2) independent researchers (A.P. and N.Z.) evaluated the eligibility of the abstracts, and two reasons arose for exclusion: (a) nine (9) duplicates were removed, and (b) one (1) study was not related to the research topic as it featured an adult sample [63].

The next step was for the researchers to retrieve the complete texts. The two independent researchers aimed to ensure that the studies met the eligibility criteria for the scoping review. There were no conflicts between the authors. As the next step, all the studies’ characteristics were gathered and presented in Table 2. The researchers used a review matrix in excel to record the extracted data and the Mendeley reference manager software, Version 2.116.1, to collect the studies and remove duplicates. After 11 articles were excluded for the abovementioned reasons, the authors finally gathered 11 studies from the search. In the current scoping review, a thematic analysis was used as we focused on identifying and interpreting patterns within data.

### 2.3. Quality Assessment for Scoping Review Study

The literature on quality assessment in scoping review studies supports the idea that it is not mandatory [71,72,73,74,75]. However, this study used the instructions in the Cochrane Handbook and included a quality assessment identifying the possibility of bias in each study [74,75,76]. Table 2 presents factors such as limitations, funding sources, and declarations of interest.

## 3. Results

### 3.1. Quality Assessment

The small sample size was a frequent limitation, as revealed in this scoping review (Table 2). Furthermore, four studies did not report relevant information for funding and support. Three of the included studies [57,59,67] were supported by the Cerebral Palsy International Research Foundation, four studies [57,58,69,70] from the Stollery Children’s Hospital Foundation, and three studies [58,69,70] by the Edmonton Oilers Community Foundation. One study [56] was supported, in part, by the National Multipurpose Research and Training Center of the National Institute of Deafness and Other Communication Disorders and by the Final Project Fund from the Graduate College at the University of Arizona. Moreover, this study is based on a doctoral dissertation at the University of Arizona. In addition, the Coleman Institute for Cognitive Disabilities supported two studies [57,58].

Regarding the declaration of interest/disclosure, no relevant information was found in two studies. Moreover, in two studies [65,68], the author reported no conflict of interest; the remaining seven declare that at least one author has a conflict of interest. All the studies were published in journals relevant to the field of the studies. Regarding the journals, three studies [56,57,58] were published in the International Journal of Speech-Language Pathology. 

### 3.2. Presentation of Studies

As shown in Table 2, of the studies published from 2012 onwards, six were published in the last 6 years. Regarding the sample’s characteristics, nine studies included only children with CP, one study included only children with Down syndrome, and one study included children with both a CP and DS diagnosis. All the studies included small sample sizes. Specifically, a higher sample number was featured in two (2) studies that recruited eight (8) children for implementation of the LSVT, and one (1) study featured seven children. In addition, 54 children with CP were involved in LSVT LOUD, and 18 with DS were, while 42 children were females, and 27 were males (one study that used LSVT LOUD as an intervention [65] did not give information about the sample’s gender). The ages of the children ranged from 3.3 to 16 years old.

Direct and indirect measures were employed to evaluate the children’s abilities. Furthermore, four studies used the Test of Children’s Speech (TOCS+) to measure how well children can make their spoken messages understood by listeners. Two studies used the Kaufman Brief Intelligence Test-2 (KBIT-2) to briefly measure verbal and nonverbal intelligence, while two used the DDK task to measure maximum speech rate (syllables per second). One study used fMRI to evaluate the children’s abilities. 

The aspects and skills assessed included functional communication, acoustic measures (auditory screening), language (expression and comprehension), speech and articulation, and voice; vocal medical status was used to verify potential vocal fold pathology through otorhinolaryngology examination (video-laryngostroboscopy); and loudness (dB SPL), duration (s), and speaking rate were derived from the maximum duration phonation task, along with diadochokinetic skills (DDK). Regarding the duration of the intervention, all the studies reported that children with CP and DS received a total dose of the LSVT LOUD. As specified in the LSVT LOUD protocol, 16 individual 60-minute therapy sessions, at a frequency of four times per week over 4 weeks, were conducted. Additionally, the data show that LSVT LOUD positively affected children with CP and DS with a diagnosis of dysarthria.

Regarding the methodology, two studies used another approach to compare the effectiveness of LSVT LOUD. Specifically, one study [64] used traditionally used speech interventions with sound theoretical motivations and another study [68] used the speech systems intelligibility treatment (SSIT). Two studies consisted of control samples of children [64,68]. One study compared children with CP and DS [57]. Most of the studies, in terms of the methodological aspects, were conducted using a consistent methodological intervention approach. All of the studies satisfied the ethical requirements as they received approval from the Research Ethics Committee.

## 4. Discussion

This scoping review had as its primary goal an overview of the literature via a search of highly regarded databases (Scopus and PubMed) and analyzed the possible effectiveness of the LSVT LOUD approach in children with voice and speech issues arising from CP and DS. Of the 11 studies, 9 only included children with CP, 1 included children with DS, and 1 more included children with both CP and DS diagnoses. 

The analysis of the studies in Table 2 indicated positive and encouraging evidence about the implementation of LSVT LOUD in the pediatric population with CP and DS diagnoses. It is a very important tool for the clinicians and all those professionals who are involved in the therapy process with children with CP and DS. Approximately 40% to 90% of children diagnosed with CP experience motor speech impairments, which have a detrimental impact on their ability to communicate clearly [8,9]. The prominent speech characteristics of dysarthria in children with CP exhibit significant variation among individuals. Nevertheless, it is frequently noticed that there are decreases in the area of vowel space, speech rate, and vocal intensity [8,9]. In addition, the motor speech characteristics of children with DS have traditionally been classified as childhood dysarthria [10]. In a study [77], out of all the participants, 97.8% fulfilled the criteria for speech disorders and the same percentage met the criteria for motor speech disorders and 37.8% had childhood dysarthria. The dysarthria subtype that was most commonly observed was ataxia. As indicated above, dysarthria is common in children with CP and DS diagnoses. 

All the studies that were examined used both formal and informal methods to evaluate the voice and speech abilities of the children. A very common measure was the parent and expert listeners’ baseline and post-treatment ratings of LSVT. More specifically, parents and expert listeners stated that there was an improvement from baseline to post-treatment for LSVT LOUD in voice, speech, and communication [55,65,66,68,70]. As it is well-understood in clinical contexts, direct assessment only occurs once [75,78]. In contrast, assessment tools parents typically fill out offer a longitudinal reflection in real-life situations, as mentioned in other studies [75,78].

Regarding the findings, the outcomes of two studies in children with CP diagnosis revealed that LSVT LOUD increased speech function and vocal loudness [sound pressure level] [64,67] and this is consistent with other studies conducted on individuals with Parkinson’s disease [44,79]. The acoustic data about voice and speech production and intelligibility in children with spastic CP showed positive results in several studies [64,65,66,68,70] and suggested that LSVT should be a promising intervention approach. 

The very recent study of Langlois et al. [57] that included CP and DS samples recorded notable statistical changes in vowel duration and acoustic vowel space in the CP group, from before therapy to 12 weeks after treatment. Additionally, an increase in acoustic vowel space was seen in five participants with DS, and this is supported by the literature. [80,81,82]. Moreover, the above results provided initial evidence of the wide-ranging impact of intensive voice treatments on the articulatory system of children with CP and DS. 

There were two studies [59,67] that an offered indication of activity-dependent neuroplasticity following LSVT LOUD in children with a CP diagnosis. Similar findings were found in the study of Narayana et al. [83], in individuals with Parkinson’s disease with increased activation in the right dorsolateral prefrontal cortex. Furthermore, in the studies of Reed et al. and Bakhtiari et al. [59,67], there was evidence of the positive performance of the brain regarding white matter and brain activity after the intensive implementation of LSVT in children with cerebral palsy [59,67]. Another study, conducted by Bakhtiari et al., examined the effects of LSVT LOUD on children with CP. The above study used graphical models and analyzed changes in connectivity between specific brain regions, namely the left supramarginal gyrus, right supramarginal gyrus, and left precentral gyrus, and the results indicated that LSVT LOUD improved the functioning of the feedback system in the speech production network [67]. Specifically, the results of the study [59] showed increased integrity in association areas following LSVT and slow-phase changes were observed in the left and right cingulum and the posterior corpus callosum in children with CP. As mentioned in the study [59], the left and right corpus callosum comprise fibers projecting from the cingulate gyrus to the medial temporal and parietal lobes. The cingulum has been linked to monitoring, encoding, correcting cognitive and motor processes, and behavioral memory. Moreover, fibers in the posterior aspects of the corpus callosum which project to the occipital and temporal lobes are integral to communication between the right and left hemispheres. The aforementioned connections improve opportunities for auditory and somatosensory feedback commands in speech production. Furthermore, as also referred in the study [59], the slow-phase changes observed in the white matter integrity of the cingulate gyrus and potentially the posterior corpus callosum in children with CP are encouraging and in line with establishing an internal calibration of appropriate vocal effort for speaking. According to the literature [84,85], LSVT LOUD follows the concept of activity-dependent neuroplasticity (e.g., sensory feedback, repetitive practice). In addition, a study [86] showed that learning to move expertly changes how the brain is wired to handle complex movements, including speech and voice. 

The study by Boliek and Fox [69] was the only study that investigated the individual and environmental aspects. The results indicated that both individual and environmental factors influenced the fast-phase and long-phase responses to LSVT LOUD. More precisely, those who respond weakly to treatment may need a longer duration of treatment, more appropriate timing in terms of their developmental and social needs, and a greater readiness to effectively communicate in their everyday activities [69]. Furthermore, those who respond well to treatment seem to gain advantages from the high level of treatment intensity and relevance, as well as from both internal and external incentives for utilizing the acquired abilities in their daily interactions [69].

Regarding the age of the children that were involved in the LSVT approach, half of studies featured children aged below 8 years old. The above is quite important because according to a study’s outcomes the period between 5 and 7 years of age is crucial for the development of connected speech. During this time, nearly all children experience notable improvements in speech intelligibility and the number of intelligible words spoken per minute [87]. Implementing approaches such as LSVT for children at an earlier stage yields improved results. 

At this point, it should be mentioned that three studies [56,58,64] reported that children with CP and DS diagnoses were able to participate in intense voice treatment without experiencing any negative effects, and this can be compared to findings of the study of Mahler and Jones [88] in two adults with dysarthria secondary to DS. Regarding the long-term sustainability and maintenance over time of the treatment effects and the improvements in the voice and speech of the children, the data that were extracted from the studies referred to the immediate period after treatment so it is not possible to present any conclusions. 

### 4.1. Key Findings

- Speech function and sound pressure level increased.
- Auditory–perceptual ratings of voice and speech revealed improvement.
- Parents’ and expert listeners’ ratings of voice, perception of vocal loudness, speech, and communication indicated improvement.
- Significant post-treatment increases in average vocal intensity during sustained vowel phonations and sentence repetitions were observed for the preschoolers.
- Increased fractional anisotropy in several motor and association tracts was observed.
- Acoustic data on untrained tasks correlated with FA changes detected.
- Post-treatment changes in connectivity between the left supramarginal gyrus, the right supramarginal gyrus, and the left precentral gyrus for children with cerebral palsy were observed.
- Treatment enhanced the contributions of the feedback system in the speech production network.
- Increased acoustic vowel space was found.
- Articulatory accuracy and single-word intelligibility improved immediately post-treatment.
- Individual and environmental features affected fast-phase and long-phase responses to LSVT LOUD.
- Intensity (dose) is necessary but not sufficient for change.
- Weak responders may require a more extended treatment phase, better timing, and a more prominent desire to communicate successfully. In contrast, strong responders benefit from treatment intensity and saliency and intrinsic and extrinsic rewards for using the trained skills for everyday communication.
- Children with DS tolerated intensive voice treatment without adverse effects and made select meaningful therapeutic gains.

### 4.2. Strengths and Limitations

In our study, we acknowledged that the first strong point of the studies was the clear methodology, and the second was that the participants were appropriately diagnosed with CP and DS. However, the study has limitations, and it is crucial to evaluate whether the intervention outcomes observed in these studies represent enough of those observed in the larger population of children with CP or DS. Furthermore, this study could not assess the possible effects of gender or other socio-demographic factors in response to the intervention because the studies did not include relative data. 

Finally, in our review study, all included studies show evidence that LSVT LOUD improves children’s skills, as indicated by their findings and conclusions. However, a study design should focus on a comparison with other therapies with a larger sample size or aim to research the long-term effects of the LSVT LOUD approach. Nevertheless, findings like these may prompt more researchers to design studies with a larger sample size of children with CP or DS. 

## 5. Conclusions

Moreover, findings revealed that the children with CP and DS showed positive reactions when involved in the full dose of LSVT, and this was clear when comparing the baseline assessment for the post-treatment evaluation. The included studies confirmed the effectiveness of LSVT for dysarthria in children with DS and CP, compared with either other speech interventions or no intervention. Parents of these children perceived benefits from intensive intervention and indicated they would recommend LSVT LOUD to other parents. However, the evidence so far cannot be generalizable due to the limited sample size. Furthermore, it would be worthwhile for the researchers and clinicians (e.g., speech and language therapists) to undertake future randomized controlled trials to implement LVST within a larger sample size of children with CP and DS, as it is essential to evaluate further the effectiveness of LSVT for dysarthria in children with CP and DS. 

In conclusion, the evidence is sufficient and conclusive based on our comprehensive analysis. Our review highlights the key finding that all included studies prove that LSVT LOUD improves children’s voice and speech skills (vowel duration, acoustic vowel space, and increased articulatory accuracy) and LSVT could be suggested as an effective intervention approach for children with CP and DS diagnosis. 

## Figures and Tables

**Table 1:** The PCC framework.

PCC Element	
Population	Children
Concept	The use of the LSVT LOUD approach
Context	Every disorder that affects children

**Table 2:** Data extraction and synthesis (sample characteristics and essential findings of the articles included in the review).

Study	Sample Gender (M/F)	Age [Range] (Years)	Disorder of the Child	Assessment	Duration	Interventions	Findings
Levy et al., 2013 [64]	2 [LSVT] 1 [Control] 3 (F)	7.03 [3.3–9.7]	CP	-Test of Auditory Comprehension of Language-3 (TACL-3). -Kaufman Brief Intelligence Test-2 (KBIT-2). -Informal assessment.	Four days per week (total 4 weeks) where the LSVT LOUD intervention was administered for 50 to 60 min plus 10 min of homework.	1. LSVT LOUD. 2. Speech intervention with sound theoretical motivations.	Speech function and sound pressure level increased in children with dysarthria, although success may vary across linguistic levels and children.
Watts 2013 [65]	5 [LSVT] 5 [Control]	[5–7]	CP	-Informal assessment.	Sixteen treatment sessions across 4 consecutive weeks, with four treatment sessions per week.	LSVT LOUD blinded (participants were randomly assigned).	Positive results for voice and speech production in children with CP. Auditory–perceptual ratings of voice and speech revealed improvement in CP. Parent’s ratings of voice, speech, and communication indicated improvement.
Fortin et al., 2023 [66]	1 (F)	5 years old	CP	-Functional communication measures of the American Speech–Language–Hearing Association (1997). -Clinical evaluation. -Assessment of vocal medical status to verify potential vocal fold pathology through otorhinolaryngologic examination (videolaryngostroboscopy). -Acoustic measures.	As specified in the LSVT LOUD protocol, the girl received 16 individual 1 h long therapy sessions four times per week over a period of 4 weeks.	Standard LSVT LOUD acoustic measures that included average vocal intensity during sustained vowel phonations and sentence repetitions and the maximum duration of sustained vowel phonations were collected pre- and post-treatment.	Significant post-treatment increases in average vocal intensity during sustained vowel phonations and sentence repetitions for the preschooler. Significant increases in the maximum duration of sustained vowel phonation pre- to post-treatment for the preschooler. Perceptual ratings revealed improvements in communicative effectiveness, participation, and speech.
Reed et al. [59]	8 [LSVT] 3 (F) 5 (M) 8 [Control]	11.6 [7–16]	CP	A pediatric neurologist made the diagnosis; the GMFCS expanded and a revised grade was determined by a physical therapist, and a licensed speech-language pathologist determined speech/voice status. -Test of Children’s Speech (TOCS+). -Loudness (dB SPL) and duration (s) were derived from the maximum duration phonation task. -Loudness, speaking rate (syllables per s), and pitch variability (F 0 in Hz) were derived from the vowel segments of the phrase repetition task. -The DDK sequential motion rate task measured the MMRtri repetition rate (syllables/s).	Each participant with CP received a total dose of LSVT LOUD provided by a certified speech-language pathologist.	-A certified speech-language pathologist provides LSVT LOUD for children with CP. -Control children received no additional speech or language activities outside their typical daily home and school routines.	Immediately following treatment and after the 12-week maintenance program the following were observed: Increased fractional anisotropy in several motor and association tracts. Acoustic data on untrained tasks correlated with FA changes.
Bakhtiari et al., 2017 [67]	8 [LSVT] 3 (F) 5 (M) 8 [Control]	11.6 [7–16]	CP with dysarthria diagnosis (with motor speech disorders)	-Test of Children’s Speech Plus (TOCS+). -The DDK task. -Pre-treatment: Children were asked to overtly produce phonation at conversational loudness, cued-phonation at perceived twice-conversational loudness, a series of single words, and a prosodic imitation task while being scanned using fMRI.	Four weeks of LSVT LOUD, followed by a 12-week maintenance program.	-Children with CP: intensive neuroplasticity-principled voice treatment protocol; LSVT LOUD. -Control children did not receive treatment or any similar training throughout the course of the study.	Post-treatment changes in connectivity between the left supramarginal gyrus, the right supramarginal gyrus, and the left precentral gyrus for children with cerebral palsy. LSVT LOUD enhanced the contributions of the feedback system in the speech production network instead of having a high reliance on the feedforward control system and the somatosensory target map for regulating vocal effort.
Levy 2014 [68]	1 (M) {LSVT] 1 (F) [SSIT]	7 13	CP with dysarthria diagnosis	Test for Auditory Comprehension of Language—3rd Edition. -Kaufman Brief Intelligence Test—2nd Edition. -Audiological screening.	A total of 1 h for 4 days per week for 4 weeks (16 day sessions for each intervention).	-LSVT. -Speech Systems Intelligibility Treatment (SSIT).	Better acoustic vowel space following LSVT LOUD and SSIT. Increased articulatory accuracy and post-treatment stimuli were preferred, and they were judged more intelligible than pre-treatment stimuli.
Boliek and Fox 2014 [69]	1 (M) 1 (F)	10:9 for both children	CP with dysarthria diagnosis	-Acoustic and perceptual data were collected according to standardized protocols. -Measurements of untrained and trained tasks. -The standardized TOCS +.	Four individual 1 h sessions, 4 days per week for 4 weeks, delivered by certified LSVT clinicians.	LSVT	Individual and environmental features affected fast-phase and long-phase responses to LSVT LOUD. Intensity (dose) is necessary but not sufficient for change. Weak responders may require a more extended treatment phase, better timing (e.g., developmentally and socially), and a more prominent desire to communicate successfully during daily activities. Strong responders appear to benefit from treatment intensity and saliency and intrinsic and extrinsic rewards for using trained skills for everyday communication.
Boliek and Fox 2017 [70]	7 (5 F, 2 Μ)	[6–10]	Spastic CP with dysarthria diagnosis	-Auditory–perceptual measures (listener task). -Acoustic measures. -Parent ratings. -Visual analog scales, perceptual ratings of single-word intelligibility, and parent interviews.	Four individual 1 h sessions, 4 days per week for 4 weeks.	LSVT	Expert listeners preferred voice quality and the articulatory precision of children with CP at follow-up compared to pre-treatment. Acoustic data indicated improvements in select measures of vocal functioning immediately post-treatment, with some maintenance at follow-up. Single-word intelligibility improved immediately post-treatment. Parents rated positive changes in voice and speech characteristics and qualitative changes in communication.
Langlois et al., 2020 [57]	17 (CP) 8 (M) 9 (F) 9 (DS) 1 (M) 8 (F)	10.6 (CP) [6–16] 6.8 (DS) [4–8]	CP and DS with dysarthria diagnosis	-Recordings. -Test of Children’s Speech Plus (TOCS+) for the CP group. -The Goldman–Fristoe Test of Articulation 2 (GFTA) for the DS group and the sentences.	CP and DS groups: 16 one-hour sessions delivered over four weeks (four days per week) and daily homework assignments (one per day on treatment days and two per day on non-treatment days).	LSVT	Significant changes in vowel duration and acoustic vowel space occurred from pre-treatment to 12 weeks post-treatment in those with CP. Increased acoustic vowel space was observed in five participants with Down syndrome. Findings provide preliminary evidence of intensive voice treatment spreading effects on the articulatory system in some children with CP and DS.
Fox and Bolek 2012 [56]	5 3 (M) 2 (F)	6.5 [5–7]	CP with dysarthria diagnosis	-Brief voice and speech screening. -An assessment of abilities to follow directions related to the study tasks. -A hearing screening (500 Hz, 1000 Hz, 2000 Hz, and 4000 Hz at 25 dB HL). -Parent rating forms. Visual analog scale.	Sixteen treatment sessions (four sessions a week for 4 consecutive weeks), two recording sessions 1 week immediately following treatment, and two recording sessions 6 weeks after the conclusion of treatment.	LSVT	Findings support that the children with CP tolerated intensive voice treatment and showed improvement in select aspects of vocal functioning. Parents reported an improved perception of vocal loudness immediately following treatment. Listeners preferred the speech samples taken immediately post-treatment over those taken during the baseline phase.
Bolek et al. 2022, [58]	9 8 (F) 1 (M)	[4;6 and 8;10]	DS with motor speech disorders (dysarthria diagnosis).	-PPVT-R. -Expressive One-Word Picture Vocabulary Test-R. -Expressive language without modeling. Ratings of single-word intelligibility.	All participants completed the total dose of LSVT LOUD: four individual 1 h sessions, 4 days per week for 4 weeks.	LSVT	Children with DS tolerated intensive voice treatment without adverse effects and made select meaningful therapeutic gains. Patterns of post-treatment improvements were not consistent across participants but were more frequently observed on trained maximum performance tasks compared to tasks reflecting the general treatment skillset. Some participants exhibited a more robust response to treatment, whereas others showed a mixed or weaker response. Parents perceived benefits from intensive intervention and indicated they would strongly recommend LSVT LOUD to other parents who have children with DS and motor speech disorders.

LSVT LOUD: Lee Silverman Voice Treatment; SPL: sound pressure level; CP: cerebral palsy; DS: Down syndrome; FA: fractional anisotropy; TOCS+: Test of Children’s Speech Plus; GFTA: Goldman–Fristoe Test of Articulation 2; PPVT-R: Peabody Picture Vocabulary Test-Revised.

## Data Availability

Not applicable.
